# U-shaped association of the non-HDL/HDL ratio with cognitive impairment identified by conventional analyses and machine learning in health examination participants in Liuyang

**DOI:** 10.3389/fnhum.2026.1775215

**Published:** 2026-02-18

**Authors:** Xiaoyi Chen, Runzhui Lin, Le Zhao, Yong He, Tieshi Zhu

**Affiliations:** 1 Department of Neurology, Zhanjiang Central Hospital, Guangdong Medical University, Zhanjiang, Guangdong, China; 2 Department of Hepatobiliary, Pancreatic and Splenic Surgery, Second Affiliated Hospital of Shantou University Medical College, Shantou, China; 3 Department of Medical Affairs, Central Hospital of Guangdong Provincial Nongken, Zhanjiang, Guangdong, China; 4 Department of Neurology, Liuyang Jili Hospital, Changsha, Hunan, China

**Keywords:** Chinese rural residents, cognitive function, cross-sectional study, lipid management, machine learning

## Abstract

**Background:**

The non-high-density lipoprotein cholesterol to high-density lipoprotein cholesterol ratio (NHHR) is associated with cardiovascular risk, but its relationship with cognitive impairment has not been well studied.

**Methods:**

In this cross-sectional study, 1,103 adults (median age, 68 years; 54.7% women) from Liuyang were included. Cognitive function was assessed by Mini-Mental State Examination. Restricted cubic splines were used to evaluate associations of NHHR with cognitive impairment. Breakpoint regression identified inflection points. Discrimination was compared using area under the curve (AUC). Machine learning with SHapley Additive exPlanations (SHAP) was applied to assess the relative importance of NHHR and to further explore its relationship with cognitive impairment.

**Results:**

Overall, 241 participants (21.9%) had cognitive impairment. NHHR demonstrated a significant U-shaped association with cognitive impairment (overall and nonlinearity *p* < 0.001). Breakpoint regression identified an inflection point at 2.772; NHHR ≥2.772 was associated with increased risk (odds ratio, 3.36; 95% CI, 2.23–5.05; *p* < 0.001). Compared with LDL, HDL, and non-HDL, NHHR had the greatest AUC for discriminating cognitive impairment. SHAP analysis confirmed the U-shaped relationship and identified NHHR as the most influential lipid-related predictor.

**Conclusion:**

In this cross-sectional analysis, NHHR was associated with cognitive impairment in a U-shaped pattern and demonstrated better discrimination than individual lipid measures. These findings suggest that NHHR may serve as an alternative lipid-related index in studies of cognitive health, although longitudinal studies are needed to clarify its predictive value.

## 1 Introduction

Cognitive impairment, particularly dementia, poses a growing global public health concern, with substantial implications for individuals, families, and healthcare systems (Lee et al., 2023; Reuben et al., 2024). In 2019, an estimated 57.4 million individuals worldwide were affected by dementia, and this number is projected to rise to 152.8 million by 2050 (GBD Dementia Forecasting Collaborators, 2022). Dementia not only substantially diminishes patients’ quality of life but also places a considerable economic burden on families and healthcare systems (Kalaria et al., 2024; Sommerlad et al., 2023). Given the current challenges in dementia treatment, identifying and addressing modifiable risk factors is crucial for developing effective prevention strategies (Budd Haeberlein et al., 2022; van Dyck et al., 2023).

Recent studies increasingly suggest a strong association between dyslipidemia and cognitive decline (Pan et al., 2024). As modifiable biological markers, lipid profiles offer promising targets for interventions aimed at preventing cognitive dysfunction (Petek et al., 2023). The non-high-density lipoprotein cholesterol to high-density lipoprotein cholesterol ratio (NHHR) has emerged as a comprehensive lipid indicator that reflects both atherogenic and protective lipoproteins (Zhen et al., 2023). NHHR has been recognized for its utility in cardiovascular risk assessment (Liu et al., 2024). Compared with traditional lipid parameters, NHHR may provide a more holistic reflection of lipid-related vascular risk, yet evidence supporting its role in cognitive health is limited. Therefore, this study aimed to investigate the association between NHHR and cognitive function using data from the Liuyang Jili Hospital Physical Examination Center. We hypothesized that NHHR would exhibit a nonlinear relationship with cognitive function, providing new insights into the potential utility of lipid ratios in strategies for cognitive health maintenance and dementia prevention.

## 2 Methods

### 2.1 Population

The study population comprised individuals from Liuyang Jili Hospital. Data were obtained from the Physical Examination Center (FY2022) and included 1,103 individuals who underwent Mini-Mental State Examination (MMSE) assessment, with a median age of 68 years.

### 2.2 Cognitive assessments

Cognitive assessment was conducted using the MMSE, which evaluates multiple cognitive domains, including orientation, memory, numerical ability, and language skills (Fiorenzato et al., 2024). Cognitive impairment was defined as an MMSE score of ≤17 for individuals with no formal education, ≤19 for those with a primary school education, and ≤24 for those with a junior high school education or higher (He et al., 2025).

### 2.3 Blood sampling and laboratory measurements

Venous blood samples were collected in the morning after an overnight fast (≥8 h). Serum was separated and analyzed in the certified clinical laboratory of Liuyang Jili Hospital. Total cholesterol (TC), triglycerides (TG), and HDL were measured using enzymatic methods on an automated clinical chemistry analyzer with manufacturer-provided reagents. Routine internal quality control was performed daily using commercial control materials, and the analyzer was calibrated regularly according to the manufacturer’s protocol. The laboratory also participated in external quality assessment/proficiency testing programs.

### 2.4 NHHR

The NHHR is calculated using the following equation (Guo et al., 2024; Qing et al., 2024):


NHHR=(TC−HDL)/HDL

### 2.5 Covariate

Covariates included age, gender, body mass index (BMI), TC, low-density lipoprotein cholesterol (LDL), high-density lipoprotein cholesterol (HDL), education level, physical activity, dietary habits, smoking status, alcohol consumption, hypertension, diabetes mellitus (DM), and ischemic stroke. Among these, TC, LDL, and HDL levels were obtained from the hospital’s Laboratory Department, while the remaining covariates were collected through self-report.

### 2.6 Statistic

Data were analyzed using R, version 4.4.1 (R Foundation for Statistical Computing). Baseline characteristics are presented as median [interquartile range (IQR)] for continuous variables and as number (percentage) for categorical variables. Restricted cubic splines (RCS) with 3 knots at the 10th, 50th, and 90th percentiles were used to evaluate nonlinear associations between the NHHR, cognitive impairment, and log-transformed MMSE scores [log(MMSE+1)]. Breakpoint regression was applied to examine the association of NHHR with cognitive impairment before and after the estimated inflection point, which was determined by model self-fitting. The RCS-derived turning point indicates the exposure range where the association starts to strengthen under a smooth functional form, whereas the breakpoint from two-piecewise (segmented) regression represents a single data-driven cut-point that optimizes model fit for a piecewise linear approximation; thus, the two estimates may differ. To compare the discriminative ability of lipid measures, logistic regression models were fitted separately for NHHR, non-high-density lipoprotein cholesterol (NHDL), LDL, and HDL. Receiver operating characteristic (ROC) curves were generated, and the areas under the curve (AUCs) were compared using the DeLong test. Covariates included age, sex, BMI, educational attainment, exercise, dietary, smoking status, alcohol use, hypertension, DM, and history of ischemic stroke. Multicollinearity was evaluated with the variance inflation factor (VIF); values greater than 5 indicated collinearity. All covariates had VIF values less than 5 (Supplementary Table S1). Two-sided *p* < 0.05 was considered statistically significant.

In addition, we used an extreme gradient boosting (XGBoost) classifier to further evaluate the predictive contribution of NHHR to cognitive outcomes. Participants were randomly split into a training set (70%) and a test set (30%). Categorical variables were one-hot encoded based on the training set design matrix to ensure consistent feature representation in the test set, and missing values were handled internally by XGBoost without imputation. The XGBoost model was trained in the training set with stratified 5-fold cross-validation to determine the optimal number of boosting rounds using early stopping (50 rounds; maximum 2,000 rounds), and the final model was refit using the selected number of iterations. The model hyperparameters were prespecified as follows: maximum tree depth = 3, learning rate (eta) = 0.06, subsample = 0.85, colsample by tree = 0.85, minimum child weight = 1, L2 regularization (lambda) = 1, and scale pos weight was set according to the ratio of negative to positive samples in the training set. NHHR, LDL, HDL, and the covariates listed above were included as predictors. Model discrimination was assessed using ROC curves and AUC with 95% confidence intervals, calibration was evaluated using calibration plots, and clinical utility was assessed using decision curve analysis. The optimal probability threshold was determined in the training set using the Youden index and then applied to the test set; sensitivity, specificity, accuracy, positive predictive value, negative predictive value, *F*_1_-score, and the selected threshold were reported. Model interpretability was assessed using SHapley Additive exPlanations (SHAP, TreeSHAP); feature importance was summarized as mean absolute SHAP values, and 95% confidence intervals were obtained using bootstrap resampling (1,000 resamples). SHAP dependency plots were further generated to examine the dependency of NHHR and its interactions with LDL and HDL.

## 3 Results

### 3.1 Baseline characteristics of the study population

A total of 1,103 participants (median age, 68 years; 54.7% women) were included, of whom 241 (21.9%) had cognitive impairment and 862 (78.1%) had normal cognition. Compared with participants with normal cognition, those with cognitive impairment had lower MMSE scores, lower HDL levels, higher NHDL levels, higher NHHR, higher educational attainment, lower rates of regular exercise, a higher prevalence of ischemic stroke, and a greater proportion of nondrinkers (*p* all <0.05). No significant differences were observed between groups in age, sex, BMI, TC, LDL, dietary, hypertension, or DM (*p* all >0.05). Smoking status showed a borderline association (*p* = 0.05) (Table 1).

**Table 1:** Baseline characteristics of participants stratified by cognitive impairment status.

Variables	Total *n* = 1,103	Yes *n* = 241	No *n* = 862	*p*
Age (year)	68.0 (66.0, 72.0)	69.0 (66.0, 74.0)	68.0 (66.0, 72.0)	0.101
Female	603 (54.7%)	139 (57.7%)	464 (53.8%)	0.323
BMI (kg/m^2^)	24.4 (22.3, 26.3)	24.7 (22.1, 26.6)	24.4 (22.3, 26.2)	0.533
MMSE	25.0 (21.0, 28.0)	19.0 (15.0, 23.0)	26.0 (24.0, 28.0)	<0.001
TC (mmol/L)	5.09 (4.32, 5.77)	5.20 (4.44, 5.85)	5.04 (4.29, 5.75)	0.055
LDL (mmol/L)	2.96 (2.36, 3.52)	3.05 (2.34, 3.59)	2.94 (2.36, 3.52)	0.749
HDL (mmol/L)	1.44 (1.23, 1.67)	1.37 (1.20, 1.60)	1.46 (1.25, 1.68)	0.004
NHDL (mmol/L)	3.59 (2.91, 4.23)	3.76 (3.00, 4.46)	3.55 (2.88, 4.16)	0.008
NHHR	2.50 (1.99, 3.05)	2.64 (2.00, 3.57)	2.47 (1.98, 2.94)	<0.001
Education				<0.001
>Elementary school	328 (29.7%)	105 (43.6%)	223 (25.9%)	
Elementary school	584 (52.9%)	98 (40.7%)	486 (56.4%)	
Illiterate	191 (17.3%)	38 (15.8%)	153 (17.7%)	
Exercise				0.003
Never	494 (44.8%)	131 (54.4%)	363 (42.1%)	
Sometime	49 (4.4%)	7 (2.90%)	42 (4.9%)	
Everyday	560 (50.8%)	103 (42.7%)	457 (53.0%)	
Diet				0.147
Unbalance	25 (2.3%)	2 (0.8%)	23 (2.7%)	
Balance	1,078 (97.7%)	239 (99.2%)	839 (97.3%)	
Smoke				0.050
Never	855 (77.5%)	195 (80.9%)	660 (76.6%)	
Former	48 (4.4%)	14 (5.8%)	34 (3.9%)	
Current	200 (18.1%)	32 (13.3%)	168 (19.5%)	
Drink				0.021
Never	996 (90.3%)	225 (93.4%)	771 (89.4%)	
Sometime	69 (6.3%)	6 (2.5%)	63 (7.3%)	
Everyday	38 (3.4%)	10 (4.1%)	28 (3.3%)	
Hypertension	545 (49.4%)	130 (53.9%)	415 (48.1%)	0.129
Diabetes	167 (15.1%)	40 (16.6%)	127 (14.7%)	0.540
Ischemic stroke	21 (1.9%)	11 (4.6%)	10 (1.2%)	0.002

Data are presented as median (Q1, Q3) or *n* (%). Q1, 1st quartile; Q3, 3rd quartile; MMSE, Mini-Mental State Examination; BMI, body mass index; TC, total cholesterol; LDL, low-density lipoprotein cholesterol; HDL, high-density lipoprotein cholesterol; NHDL, non-HDL; NHHR, non-high-density lipoprotein cholesterol to high-density lipoprotein cholesterol ratio. Cognitive impairment was defined using education-stratified MMSE cutoffs: MMSE ≤17 for illiterate participants, ≤19 for primary school education, and ≤24 for junior high school education or above.

### 3.2 NHHR has U-shaped relationship with risk of cognitive impairment

**Figure 1:**
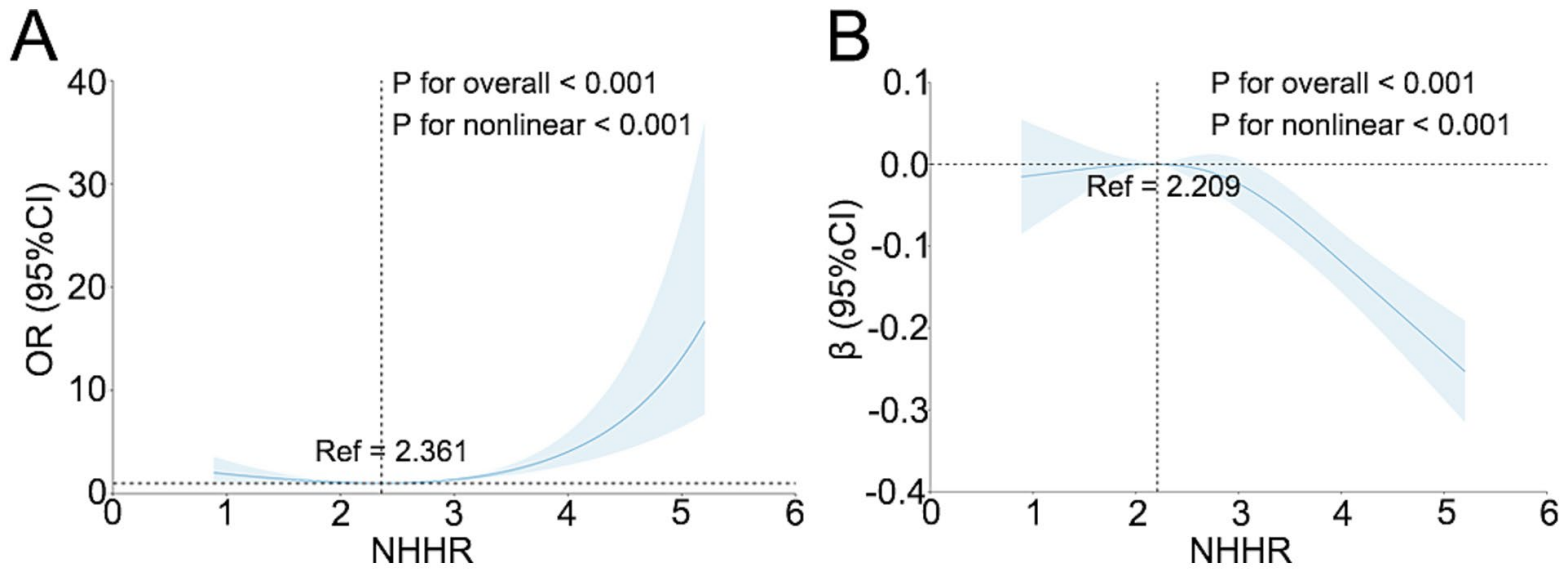
Restricted cubic spline analysis of the association of NHHR with cognitive impairment and log(MMSE+1). **(A)** Adjusted association of NHHR with cognitive impairment based on restricted cubic spline models. **(B)** Adjusted association of NHHR with log(MMSE+1) based on restricted cubic spline models. Solid lines indicate fitted values, and shaded areas indicate 95% confidence intervals.

**Figure 2:**
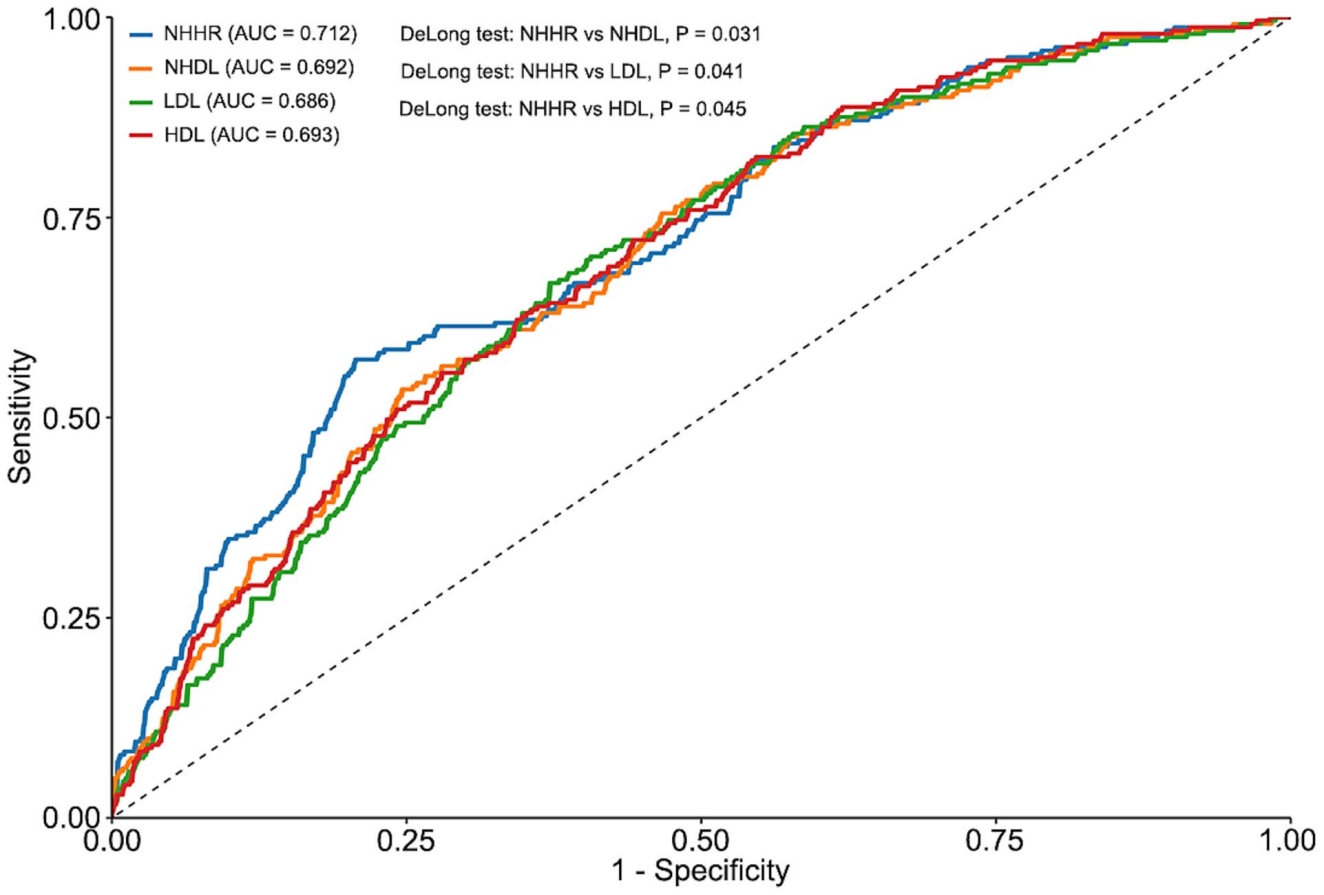
Receiver operating characteristic curves comparing NHHR, NHDL, LDL, and HDL for predicting cognitive impairment. Receiver operating characteristic (ROC) curves for NHHR, NHDL, LDL, and HDL in predicting cognitive impairment. The area under the curve (AUC) for each marker is shown. *p*-values were obtained using DeLong’s test for differences between AUCs.

**Figure 3:**
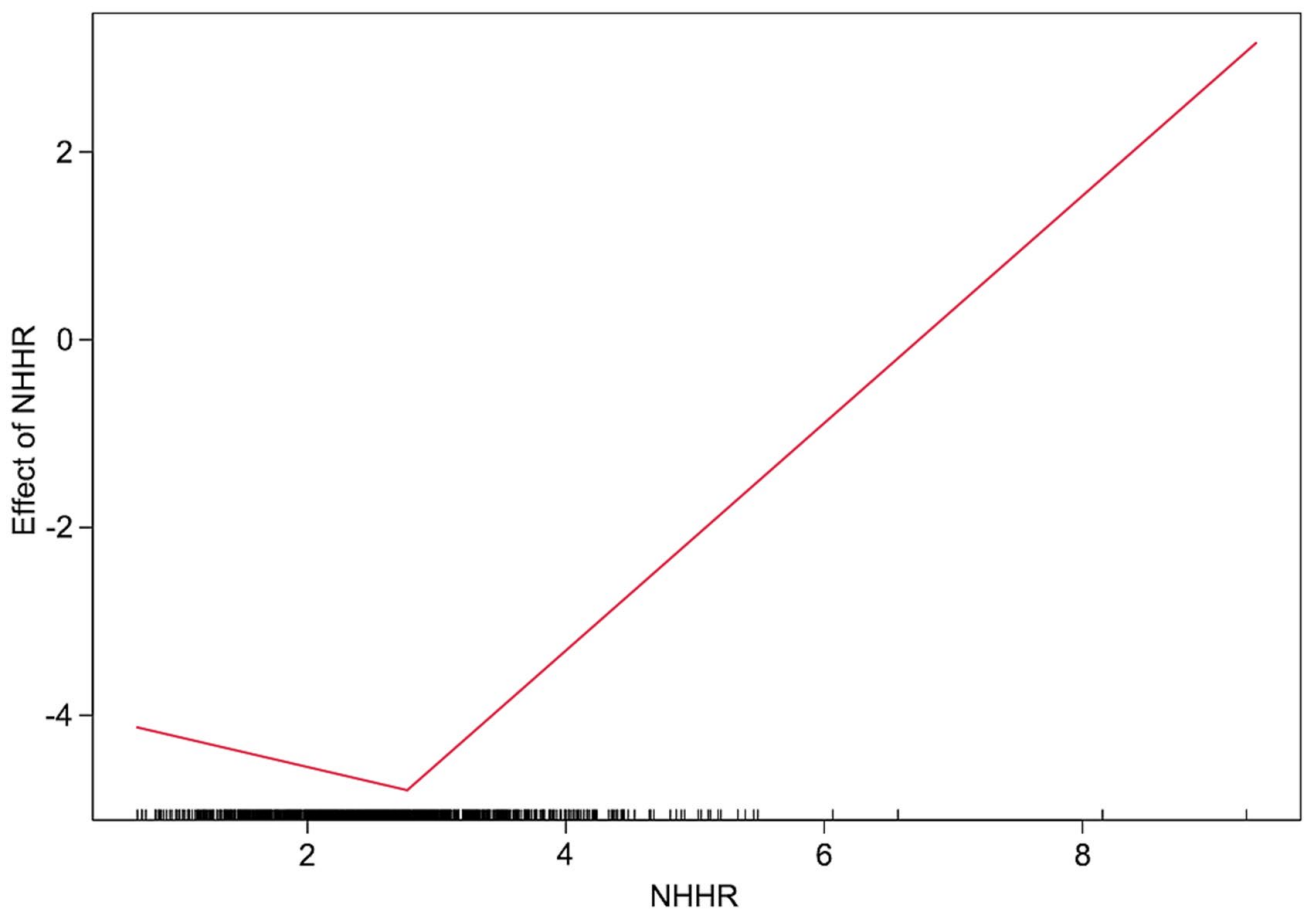
Breakpoint regression analysis of the association between NHHR and cognitive impairment. The fitted curve from breakpoint regression analysis showing the association between NHHR and the risk of cognitive impairment. A breakpoint was identified at NHHR = 2.772. The solid line represents the estimated effect of NHHR, and tick marks along the *x*-axis represent the distribution of participants.

RCS analyses demonstrated significant nonlinear associations of NHHR with both cognitive impairment and cognitive function (overall *p* < 0.001; nonlinearity *p* < 0.001), with U-shaped curves and turning points near 2.361 and 2.209, respectively (Figures 1A,B). In AUC comparisons from logistic regression models, NHHR showed the greatest discriminative performance for cognitive impairment compared with NHDL, LDL, and HDL (Figure 2). Breakpoint regression further supported a U-shaped association, identifying a best-fitting threshold at NHHR = 2.772. Below this threshold, NHHR was not significantly associated with cognitive impairment [odds ratio (OR), 0.73; 95% CI, 0.48–1.09; *p* = 0.124]; at or above the threshold, higher NHHR was associated with substantially increased risk (OR, 3.36; 95% CI, 2.23–5.05; *p* < 0.001) (Table 2 and Figure 3).

**Table 2:** Breakpoint regression analysis of the association between NHHR and cognitive impairment.

Variables	OR	95% CI	*p*
NHHR <2.772	0.726	(0.482, 1.092)	0.124
NHHR ≥2.772	3.357	(2.233, 5.047)	<0.001

NHHR, non-high-density lipoprotein cholesterol to high-density lipoprotein cholesterol ratio.

### 3.3 Machine learning analysis

**Figure 4:**
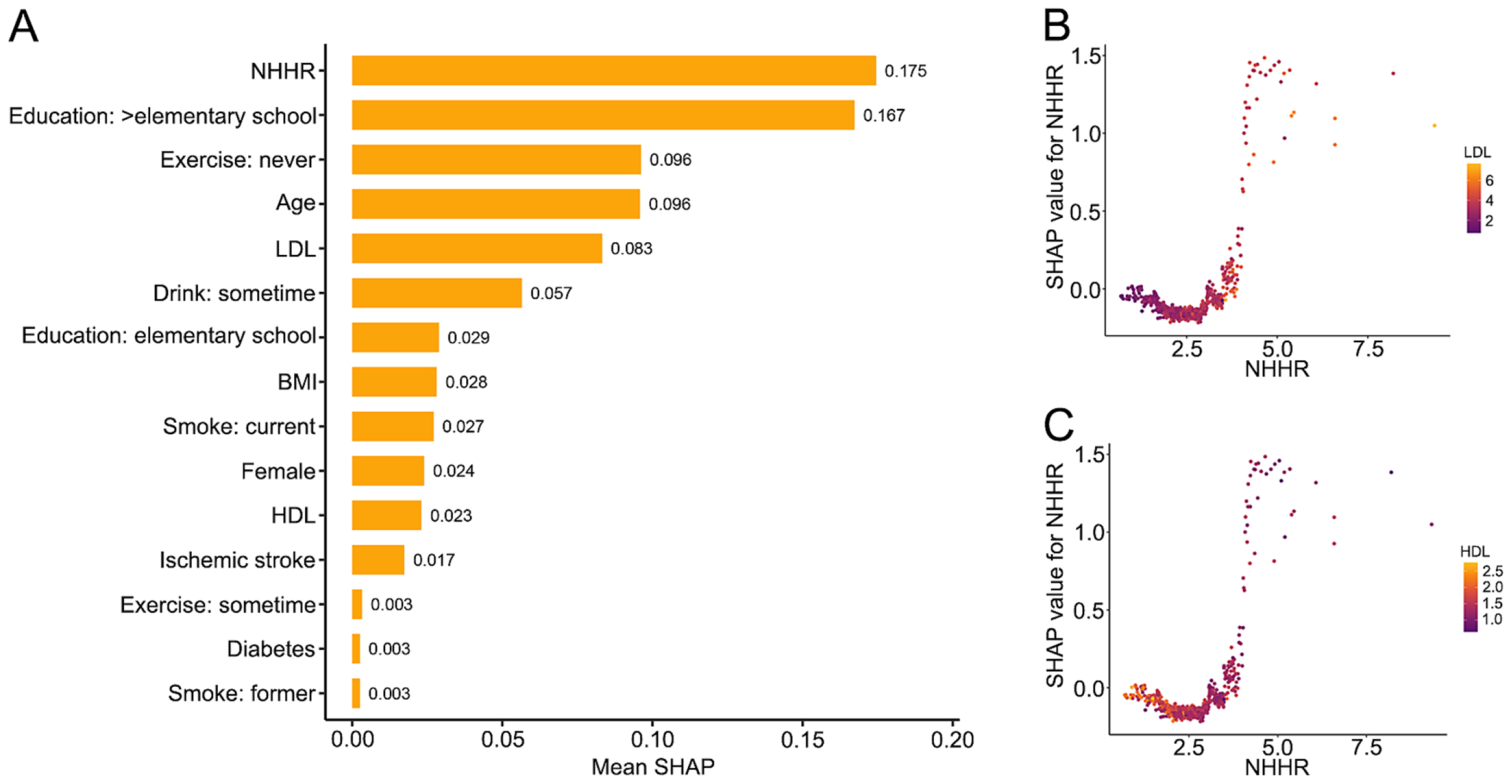
SHAP analysis of the contribution of NHHR and other variables to cognitive impairment prediction. **(A)** Ranking of variables by mean SHAP values, showing their relative importance in predicting cognitive impairment. **(B)** SHAP dependence plot of NHHR with LDL levels overlaid. **(C)** SHAP dependence plot of NHHR with HDL levels overlaid. Each point represents an individual participant.

The study population was randomly divided into a training dataset (*n* = 773) and a test dataset (*n* = 330), with no significant differences in baseline clinical characteristics between groups (Supplementary Table S2). An XGBoost model was developed in the training dataset and internally validated in the test dataset. Model performance was acceptable, as demonstrated by receiver operating characteristic curves, calibration plots, and related metrics (Supplementary Figures S1A,B,D,E and Supplementary Table S3). Decision curve analysis indicated clinical utility within specific threshold probabilities (Supplementary Figures S1C,F). SHAP analysis identified NHHR as the variable with the highest mean SHAP value, exceeding those of LDL and HDL (Figure 4A and Supplementary Table S4). Moreover, SHAP dependence plots again revealed a U-shaped association between NHHR and cognitive impairment, with the lowest SHAP values observed at NHHR around 2.5. SHAP values increased at higher NHHR levels, with greater values in the presence of elevated LDL and lower values with higher HDL (Figures 4B,C).

## 4 Discussion

In this cross-sectional study of a community-based population in China, we identified a U-shaped association between the NHHR and cognitive impairment. Breakpoint regression analysis suggested an inflection point at 2.772, above which NHHR was significantly associated with increased risk of cognitive impairment. Machine learning analysis confirmed this U-shaped relationship and indicated that NHHR contributed more strongly to the model than individual lipid markers. Collectively, these findings suggest that NHHR may better reflect lipid-related risk for cognitive impairment than single lipid measures and support its potential role as an alternative index in studies of cognitive health.

NHHR reflects the ratio of NHDL cholesterol to HDL cholesterol and is inversely related to HDL levels while positively correlated with NHDL levels. Both lipoproteins have been shown to be strongly associated with cognitive function, with HDL cholesterol demonstrating a U-shaped relationship with cognitive outcomes. For example, a recent study reported that individuals with HDL cholesterol levels exceeding 80 mg/dL had a 27% higher risk of dementia compared with those with HDL levels of 40–60 mg/dL (Hussain et al., 2024). Similarly, another study found that HDL levels above 65 mg/dL were associated with a 15% increased risk of dementia compared with the median level (53.7 mg/dL), while HDL levels between 11 and 41 mg/dL were linked to a 7% increased risk (Ferguson et al., 2023). Collectively, these findings suggest that both excessively high and low HDL cholesterol levels may increase dementia risk, aligning with the U-shaped association observed in the present study. In contrast, NHDL cholesterol primarily comprises LDL and remnant cholesterol (RC), both of which, when elevated, have been associated with cognitive decline. A large population-based study from the United Kingdom found that a 1-standard deviation increase in LDL cholesterol (39 mg/dL) was associated with a 5% increased risk of dementia in the general population. Additionally, higher concentrations of RC were linked to greater risks of all-cause dementia, Alzheimer’s disease, and vascular dementia. Compared with participants in the lowest quartile of RC, those in the highest quartile had hazard ratios of 1.11 (95% CI, 1.09–1.13) for all-cause dementia, 1.11 (95% CI, 1.08–1.13) for Alzheimer’s disease, and 1.15 (95% CI, 1.09–1.21) for vascular dementia. Notably, a recent cross-sectional study also reported a U-shaped relationship between NHDL cholesterol and cognitive function (Li et al., 2024). Taken together, these findings suggest that the right side of the U-shaped curve observed in the present study may reflect the adverse effects of elevated NHDL cholesterol and reduced HDL cholesterol, while the left side of the curve may be attributable to the detrimental impact of excessively high HDL cholesterol levels.

Our study identified a U-shaped association between NHHR and cognitive function, which may be explained by several potential biological mechanisms. At higher NHHR levels, reflecting an increased burden of NHDL lipoproteins, there is a greater propensity for the development of atherosclerosis, cerebral microvascular injury, chronic cerebral hypoperfusion, blood–brain barrier disruption, intracerebral inflammation, and oxidative stress (Bolanle et al., 2025; Chen et al., 2024; Chung et al., 2023; Dias et al., 2015; Huang et al., 2019; Lara-Guzman et al., 2018). These pathophysiological processes can ultimately result in neuronal damage and cognitive decline. In parallel, a decrease in HDL levels may weaken its protective functions, including anti-inflammatory, antioxidant, and β-amyloid clearance activities, thereby further exacerbating the risk of cognitive impairment (Grao-Cruces et al., 2022; Tziomalos et al., 2019; Zhang et al., 2022). Conversely, low NHHR is often accompanied by elevated HDL levels; however, excessively high HDL does not necessarily confer additional neuroprotection. Emerging evidence suggests that dysfunctional HDL particles may be enriched with lipids, exhibit reduced antioxidant and anti-inflammatory capacity, and display impaired reverse cholesterol transport. Such dysfunctional HDL may result in insufficient cholesterol delivery to the brain, negatively impacting neuronal synaptic plasticity and neurotransmitter synthesis (Kjeldsen et al., 2021, 2022; Suleiman et al., 2020). Additionally, reduced NHDL cholesterol levels may reflect suboptimal metabolic or nutritional conditions, which could impair brain homeostasis through insufficient provision of substrates required for neuronal maintenance and repair processes (An et al., 2019; Gong et al., 2022; Li et al., 2020). Moreover, extreme deviations in NHHR may disrupt reverse cholesterol transport. At very low NHHR levels, although HDL concentrations are high, its functional capacity to accept and transport cholesterol may be diminished. Conversely, at very high NHHR levels, the excessive accumulation of NHDL lipoproteins may overwhelm the clearance capacity of HDL, leading to cholesterol retention within the arterial wall, thereby promoting the progression of atherosclerotic plaque (Kjeldsen et al., 2021; Bhale et al., 2024; Durrington et al., 2025; Tosheska Trajkovska and Topuzovska, 2017). In summary, elevated NHHR likely contributes to cognitive impairment through mechanisms related to vascular injury and inflammation, whereas low NHHR is more likely linked to HDL dysfunction and cerebral cholesterol deficiency. Both extremes may disrupt cognitive health, underscoring the potential importance of maintaining NHHR within an appropriate range for preserving cognitive function.

The present study found that cognitive function appeared to be optimal when NHHR was maintained within the range of approximately 2.8, a finding that is generally consistent with existing lipid management guidelines (Patel et al., 2025). When NHDL cholesterol is <130 mg/dL and HDL cholesterol is >40 mg/dL, NHHR naturally falls within this range. Maintaining NHHR within the range of approximately 2.8 provides clinicians with a simple and intuitive target for lipid management. Compared to focusing solely on LDL or HDL levels, NHHR offers a more comprehensive assessment of lipid metabolism, facilitating the development of more individualized intervention strategies.

This study has several limitations. First, as a cross-sectional study, it can only establish associations rather than causality. Second, although multiple confounding variables were adjusted for, our analysis did not account for potential residual confounders such as APOE genotype and psychological factors including depression and anxiety, which are known to influence cognitive function. Third, lipid levels were assessed based on a single measurement, whereas lipid profiles may fluctuate over time; dynamic lipid monitoring may provide a more accurate reflection of long-term lipid status. Fourth, the study population was derived from a hospital-based sample, which may introduce selection bias and limit the generalizability of findings to the broader community population. Survival bias may also be present, particularly in older adults, as individuals with severe comorbidities or cognitive impairment may be underrepresented due to early mortality or inability to participate in examinations.

## 5 Conclusion

In this community-based cross-sectional study, NHHR demonstrated a significant U-shaped association with cognitive impairment, with risk increasing when NHHR exceeded an inflection point of approximately 2.8. Compared with single lipid measures, NHHR appeared to provide superior discrimination of cognitive impairment. These findings suggest that NHHR may serve as a useful lipid-related index in studies of cognitive health, although further longitudinal research is needed to clarify its predictive value and clinical implications.

## Data Availability

The data analyzed in this study is subject to the following licenses/restrictions: the data used in this study were obtained from the Liuyang Jili Hospital Medical Examination Center, and their use was contingent upon obtaining ethical approval from the hospital. Without this approval, the data could not be used, and therefore cannot be made publicly available. The authors do not have permission to share data. Requests to access these datasets should be directed to YH, 277475748@qq.com.

## Supplementary material

SUPPLEMENTARY FIGURE S1 Performance of the prediction model in training and test sets. **(A–C)** Model performance in the training set: **(A)** receiver operating characteristic (ROC) curve with the area under the curve (AUC), **(B)** calibration curve comparing predicted and observed probabilities, and **(C)** decision curve analysis (DCA) showing net clinical benefit. **(D–F)** Model performance in the test set: **(D)** ROC curve, **(E)** calibration curve, and **(F)** DCA.
