# Supplementary figures and images for: U-shaped association of the non-HDL/HDL ratio with cognitive impairment identified by conventional analyses and machine learning in health examination participants in Liuyang
Source: Front Hum Neurosci. 2026 Feb 18;20:1775215. doi: 10.3389/fnhum.2026.1775215 (PMC12957223; doi:10.3389/fnhum.2026.1775215)

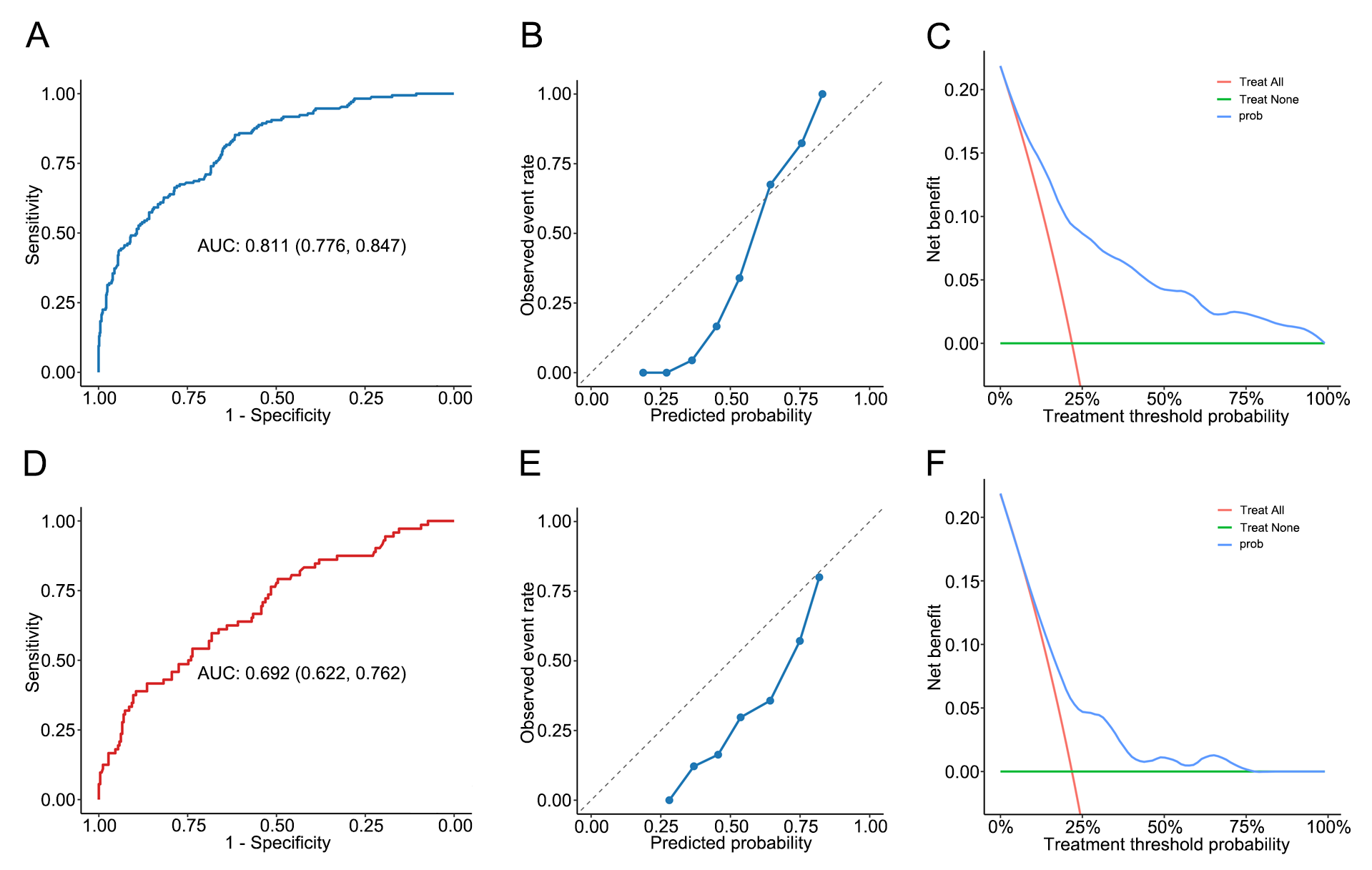

Supplement: SUPPLEMENTARY FIGURE S1 — Performance of the prediction model in training and test sets. (A–C) Model performance in the training set: (A) receiver operating characteristic (ROC) curve with the area under the curve (AUC), (B) calibration curve comparing predicted and observed probabilities, and (C) decision curve analysis (DCA) showing net clinical benefit. (D–F) Model performance in the test set: (D) ROC curve, (E) calibration curve, and (F) DCA. [file Image_1.TIF]
