# Supplementary material for: U-shaped association of the non-HDL/HDL ratio with cognitive impairment identified by conventional analyses and machine learning in health examination participants in Liuyang
Source: Front Hum Neurosci. 2026 Feb 18;20:1775215. doi: 10.3389/fnhum.2026.1775215 (PMC12957223; doi:10.3389/fnhum.2026.1775215)
Supplement: Supplementary file 2 [file Table_1.DOCX]

Table S1. VIF and Tolerance for Covariates

|  | VIF | Tolerance |
| --- | --- | --- |
| Age (year) | 1.052 | 0.951 |
| Sex |  |  |
| female | ref | ref |
| male | 1.579 | 0.633 |
| BMI (kg/m^2^) | 1.161 | 0.861 |
| Education |  |  |
| >elementary school | ref | ref |
| elementary school | 1.222 | 0.819 |
| illiterate | 1.243 | 0.806 |
| Exercise |  |  |
| everyday | ref | ref |
| never | 1.063 | 0.941 |
| sometime | 1.046 | 0.956 |
| Diet |  |  |
| balance | ref | ref |
| unbalance | 1.013 | 0.987 |
| Smoke |  |  |
| current | ref | ref |
| former | 1.383 | 0.723 |
| never | 1.863 | 0.537 |
| Drink |  |  |
| everyday | ref | ref |
| never | 1.992 | 0.502 |
| sometime | 1.897 | 0.527 |
| Hypertension |  |  |
| no | ref | ref |
| yes | 1.059 | 0.945 |
| Diabetes |  |  |
| no | ref | ref |
| yes | 1.062 | 0.942 |
| Ischemic stroke |  |  |
| no | ref | ref |
| yes | 1.035 | 0.966 |
| LDL | 3.867 | 0.259 |
| HDL | 3.761 | 0.266 |

VIF, variance inflation factor; BMI, body mass index; LDL, low-density lipoprotein cholesterol; HDL, high-density lipoprotein cholesterol. “ref” denotes the reference category for categorical variables.
