# Supplementary material for: U-shaped association of the non-HDL/HDL ratio with cognitive impairment identified by conventional analyses and machine learning in health examination participants in Liuyang
Source: Front Hum Neurosci. 2026 Feb 18;20:1775215. doi: 10.3389/fnhum.2026.1775215 (PMC12957223; doi:10.3389/fnhum.2026.1775215)
Supplement: Supplementary file 3 [file Table_2.DOCX]

Table S2. Baseline Characteristics of Participants in the Training and Test Sets

|  | Total  n = 1103 | Test  n = 330 | Train  n = 773 | ***P*** |
| --- | --- | --- | --- | --- |
| Age (year) | 68.0 (66.0,72.0) | 69.0 (66.0,72.0) | 68.0 (66.0,73.0) | 0.985 |
| Female | 603 (54.7%) | 183 (55.5%) | 420 (54.3%) | 0.782 |
| BMI (kg/m^2^) | 24.4 (22.3,26.3) | 24.4 (22.4,26.3) | 24.4 (22.2,26.4) | 0.907 |
| MMSE | 25.0 (21.0,28.0) | 25.0 (21.0,27.8) | 25.0 (21.0,28.0) | 0.754 |
| Cognitive impairment | 241 (21.8%) | 72 (21.8%) | 169 (21.9%) | 0.997 |
| TC (mmol/L) | 5.09 (4.32,5.77) | 5.18 (4.38,5.81) | 5.05 (4.26,5.76) | 0.165 |
| LDL (mmol/L) | 2.96 (2.36,3.52) | 3.01 (2.42,3.56) | 2.91 (2.31,3.52) | 0.196 |
| HDL (mmol/L) | 1.44 (1.23,1.67) | 1.46 (1.23,1.67) | 1.43 (1.24,1.67) | 0.74 |
| NHDL (mmol/L) | 3.59 (2.91,4.23) | 3.68 (2.94,4.27) | 3.56 (2.88,4.21) | 0.130 |
| NHHR | 2.50 (1.99,3.05) | 2.53 (1.99,3.12) | 2.50 (1.99,2.99) | 0.265 |
| Education |  |  |  | 0.837 |
| >elementary school | 328 (29.7%) | 102 (30.9%) | 226 (29.2%) |  |
| elementary school | 584 (52.9%) | 173 (52.4%) | 411 (53.2%) |  |
| illiterate | 191 (17.3%) | 55 (16.7%) | 136 (17.6%) |  |
| Exercise |  |  |  | 0.072 |
| never | 494 (44.8%) | 137 (41.5%) | 357 (46.2%) |  |
| sometime | 49 (4.4%) | 10 (3.0%) | 39 (5.0%) |  |
| everyday | 560 (50.8%) | 183 (55.5%) | 377 (48.8%) |  |
| Diet |  |  |  | 0.993 |
| unbalance | 25 (2.3%) | 8 (2.42%) | 17 (2.20%) |  |
| balance | 1078 (97.7%) | 322 (97.6%) | 756 (97.8%) |  |
| Smoke |  |  |  | 0.386 |
| never | 855 (77.5%) | 251 (76.1%) | 604 (78.1%) |  |
| former | 48 (4.4%) | 12 (3.6%) | 36 (4.7%) |  |
| current | 200 (18.1%) | 67 (20.3%) | 133 (17.2%) |  |
| Drink |  |  |  | 0.408 |
| never | 996 (90.3%) | 299 (90.6%) | 697 (90.2%) |  |
| sometime | 69 (6.3%) | 17 (5.2%) | 52 (6.7%) |  |
| everyday | 38 (3.4%) | 14 (4.2%) | 24 (3.1%) |  |
| Hypertension | 545 (49.4%) | 165 (50.0%) | 380 (49.2%) | 0.849 |
| Diabetes | 167 (15.1%) | 50 (15.2%) | 117 (15.1%) | 1.000 |
| Ischemic stroke | 21 (1.9%) | 5 (1.52%) | 16 (2.07%) | 0.706 |

Data are presented as median (Q1, Q3) or n (%). Q1, 1st Quartile; Q3, 3st Quartile; MMSE, Mini-Mental State Examination; BMI, body mass index; TC, total cholesterol; LDL, low-density lipoprotein cholesterol; HDL, high-density lipoprotein cholesterol; NHDL, non-HDL; NHHR, non–high-density lipoprotein cholesterol to high-density lipoprotein cholesterol ratio.
