# Supplementary material for: U-shaped association of the non-HDL/HDL ratio with cognitive impairment identified by conventional analyses and machine learning in health examination participants in Liuyang
Source: Front Hum Neurosci. 2026 Feb 18;20:1775215. doi: 10.3389/fnhum.2026.1775215 (PMC12957223; doi:10.3389/fnhum.2026.1775215)
Supplement: Supplementary file 4 [file Table_3.DOCX]

Table S3. Performance of the Prediction Model in Training and Test Sets

|  | Sensitivity | Specificity | Accuracy | PPV | NPV | F1 | Threshold |
| --- | --- | --- | --- | --- | --- | --- | --- |
| Train | 0.852 | 0.616 | 0.668 | 0.383 | 0.937 | 0.528 | 0.457 |
| Test | 0.778 | 0.500 | 0.561 | 0.303 | 0.890 | 0.436 | 0.457 |

PPV, positive predictive value; NPV, negative predictive value.
