# Supplementary material for: U-shaped association of the non-HDL/HDL ratio with cognitive impairment identified by conventional analyses and machine learning in health examination participants in Liuyang
Source: Front Hum Neurosci. 2026 Feb 18;20:1775215. doi: 10.3389/fnhum.2026.1775215 (PMC12957223; doi:10.3389/fnhum.2026.1775215)
Supplement: Supplementary file 5 [file Table_4.DOCX]

Table S4. Feature importance quantified by mean absolute SHAP values with bootstrap 95% confidence intervals.

| Feature | Mean SHAP value | 95%CI |
| --- | --- | --- |
| NHHR | 0.175 | 0.158-0.193 |
| Education: >elementary school | 0.167 | 0.164-0.170 |
| Exercise: never | 0.096 | 0.094-0.099 |
| Age | 0.096 | 0.087-0.104 |
| LDL | 0.083 | 0.078-0.088 |
| Drink: sometime | 0.057 | 0.048-0.065 |
| Education: elementary school | 0.029 | 0.027-0.031 |
| BMI | 0.028 | 0.026-0.030 |
| Smoke: current | 0.027 | 0.025-0.029 |
| Female | 0.024 | 0.023-0.025 |
| HDL | 0.023 | 0.022-0.025 |
| Ischemic stroke | 0.017 | 0.014-0.020 |
| Exercise: sometime | 0.003 | 0.003-0.004 |
| Diabetes | 0.003 | 0.002-0.004 |
| Smoke: former | 0.003 | 0.002-0.004 |

SHAP, Shapley additive explanation; NHHR, non–high-density lipoprotein cholesterol to high-density lipoprotein cholesterol ratio; LDL, low-density lipoprotein cholesterol; BMI, body mass index; HDL, high-density lipoprotein cholesterol.
